# Mesenchymal Stem Cells, Bioactive Factors, and Scaffolds in Bone Repair: From Research Perspectives to Clinical Practice

**DOI:** 10.3390/cells10081925

**Published:** 2021-07-29

**Authors:** Sandra Stamnitz, Aleksandra Klimczak

**Affiliations:** Laboratory of Biology of Stem and Neoplastic Cells, Hirszfeld Institute of Immunology and Experimental Therapy, Polish Academy of Sciences, 53-114 Wroclaw, Poland; sandra.gromolak@hirszfeld.pl

**Keywords:** stem cell therapy, biomaterials, bone tissue engineering, osteogenic differentiation

## Abstract

Mesenchymal stem cell-based therapies are promising tools for bone tissue regeneration. However, tracking cells and maintaining them in the site of injury is difficult. A potential solution is to seed the cells onto a biocompatible scaffold. Construct development in bone tissue engineering is a complex step-by-step process with many variables to be optimized, such as stem cell source, osteogenic molecular factors, scaffold design, and an appropriate in vivo animal model. In this review, an MSC-based tissue engineering approach for bone repair is reported. Firstly, MSC role in bone formation and regeneration is detailed. Secondly, MSC-based bone tissue biomaterial design is analyzed from a research perspective. Finally, examples of animal preclinical and human clinical trials involving MSCs and scaffolds in bone repair are presented.

## 1. Introduction

Bone tissue disorders affect millions of people worldwide and are one of the major clinical cases in orthopedics. The most common causes of bone defects are accidental injuries, skeletal diseases, osteoporosis-related fractures, tumor resections, congenital bone deformations, and aging [1]. Moreover, due to comorbidities such as diabetes, there is an up to six times greater risk to suffer a fracture and twice as slow healing rate. It is estimated that 10–20% of failed fractured bone treatments end in delayed union or even nonunion despite all modern treatment methods [2]. Additionally, fracture healing disruptions can lead to critical-sized bone defects, over 2 cm long, affecting more than half of the bone diameter [3]. These orthopedic complications remain the most challenging problem in surgery. Patients with complicated bone disorders are disabled and experience a lowered quality of life. Moreover, months of immobility can result in other health issues, including stroke, heart attacks, pressure ulcers, increased risk of infections, muscle and bone loss, and depression. Bone fracture treatment in the United States generates some of the highest costs, resulting in a significant healthcare burden [4].

Currently, the gold standard for the repair of large bone tissue defects is an autograft or allograft. However, while both autografts and allografts are the major substitutes for large bone defects, they have certain drawbacks. An autograft is restricted because of limited bone resource and donor-site morbidity. On the other hand, allografts are readily available, but may cause an immunogenic rejection [1,5]. Therefore, to address these limitations, new approaches are investigated. A promising alternative treatment of clinically challenging bone defects is bone tissue engineering.

Research on the potential beneficial effects of different components used in bone tissue engineering, including mesenchymal stem cells (MSCs), biomaterial scaffolds, and growth factors involved in osteogenesis, may provide a new therapeutic opportunity for critical-sized bone defects and non-union treatment [3]. Thus, this review aims to summarize the newest findings on the role of MSC therapies supporting a scaffold in bone regeneration.

## 2. The Role of MSCs in Bone Formation and Healing

Knowledge about bone formation and bone healing is necessary to determine effective MSC-based treatment strategies. Only a full understanding of the mechanisms underlying osteogenesis and bone healing enables the potential use of new, innovative, and most importantly, effective and safe alternatives to the traditional treatment methods, which are insufficient. MSCs are important regulators of bone modeling and remodeling as well as bone fracture repair [6].

### 2.1. Bone Ossification

Osteogenesis is a long process, lasting from the sixth or seventh week of embryonic development to about the age of 25 years. Bone ossification is classified into two types: intramembranous and endochondral. Both begins with MSCs as the precursors for different types of bone cells.

Intramembranous bone formation is responsible for developing most of the cranial bones, flat bones of the skull, and clavicle. This process begins with the differentiation of MSCs into specialized bone-forming osteoblasts, which then group into clusters of ossification centers. Osteoblasts secrete an unmineralized matrix consisting of collagen and proteoglycans, called the osteoid. It can bind calcium, which results in the hardening of the matrix and osteoblast entrapping, followed by osteoblast to osteocyte differentiation. Osteocytes are the most abundant in the bone and regulate bone remodeling. Osteoblasts are surrounded by blood vessels, forming the trabecular/spongy bone, whereas mesenchymal cells form the periosteum, a membrane on the bone surface, and differentiate into osteoblasts, secreting osteoid parallel to the existing one. Thus, new layers are created, called the compact bone, and red marrow is formed by blood vessels [7].

Endochondral ossification forms the axial skeleton and long bones. This process begins in the same manner as the intramembranous process, specifically, with MSCs as the precursors. However, the MSCs do not differentiate directly into bone cells, but intermediately into chondrocytes, secreting the extracellular matrix, and consequently, forming the cartilage model for the bone. Chondrocytes increase rapidly in number, and the matrix is mineralized, resulting in reduced availability of nutrients for the chondrocytes and their apoptosis. Next, the blood vessels start to invade the spaces left by the dead cells and bring stem cells, which differentiate into osteoblasts, responsible for bone deposition, and osteocytes [8].

### 2.2. Bone Repair

Bone healing is a complex physiological process that engages many different cell types, cytokines, chemokines, growth factors, and cellular responses. As a result, the bone is reconstituted without the scar tissue formation [9]. As with bone ossification, MSCs also play a significant role here. The process of skeletal renewal, also known as bone remodeling, is carried out by key grouping cells. These cells are primarily osteocytes, osteoblasts, osteoclast, and MSCs. The MSCs give rise to osteoblasts through osteogenic differentiation. Furthermore, they migrate to the surface of the bone fracture and modulate the microenvironment for other types of cells [10].

There are two types of bone healing: primary and secondary. The former concerns small fracture gap healing, where no movement occurs at the fracture site. A characteristic feature of primary bone healing is that no callus is formed. Instead, the process resembles normal bone remodeling, i.e., the last stage of secondary bone healing. Nevertheless, primary bone repair is rather rare, with most cases involving secondary healing [9].

Secondary fracture healing consists of four stages: (i) formation of fracture hematoma, (ii) formation of soft callus, (iii) formation of hard callus, and (iv) remodeling.

The first and probably the most important determinant of the bone healing effect is the fracture hematoma. The blood vessels immediately surround the damaged bone site following a fracture in the inflammatory phase. The repair begins with the subsequent infiltration of the inflammatory cells, which prevent infection and secrete pro-inflammatory cytokines. These chemoattractants recruit other immune cells, MSCs, and endothelial cells [11,12]. The cytokines occurring in the injured site include the bone morphogenetic proteins (BMPs), tumor necrosis factor alpha (TNF-α), vascular endothelial growth factor (VEGF), and interleukin 17 (IL-17), which has a dual role of stimulating bone resorption through osteoclasts and enhancing osteogenic efficiency through osteoblasts [9]. BMPs enhance the differentiation of MSCs into osteogenic cells, whereas VEGF stimulates vascular cells. The final step of hematoma before the next stage of fracture healing, is the generation of the extracellular matrix, which develops into the granulation tissue. This tissue consists primarily of MSCs and endothelial and immune cells [11].

Formation of the soft callus is the second stage of fracture healing, which takes place when the MSCs in the granulation tissue start to differentiate into chondroblasts, fibroblasts, and osteoblasts. The fibrocartilaginous callus is formed during endochondral ossification and connects the ends of the bone in the fracture gap [13].

Hard callus, called the woven bone, forms in the next stage following the formation of the soft callus. However, depending on stability of the fracture site, it may develop directly from the granulation tissue through intramembranous ossification. Osteoblasts produce vesicles with calcium phosphate complexes into the matrix, which in turn causes the formation of a hard, calcified callus [9].

Bone remodeling, the final stage of bone healing, can last from months to many years. The process involves joining the soft callus formed during endochondral ossification with the hard callus of intramembranous ossification, resulting in the regeneration of the weight bearing bone [12]. Osteoclasts and osteoblasts are particularly involved in this stage of repair. They create a balance between resorption and new bone formation. Vasculature also undergoes a substantial remodeling [13]. Bone resorption by the osteoclasts leads to MSC recruitment. These, in turn, create a specific microenvironment to enable bone formation [10].

## 3. MSC-Based Tissue Engineering Therapies in Bone Repair from a Research Perspective

MSCs were described in 1966 by Friedenstein et al. as cells with fibroblast-like morphology residing in the bone marrow and able to form the ectopic bone. Following the suggestion that MSCs are osteogenic precursors [14], researchers in the field of regenerative medicine began taking interest in them. However, the term “mesenchymal stem cells” was proposed by Caplan in 1991 to describe a type of adult stem cells characterized by a multipotential differentiation ability into the osteogenic, chondrogenic, and adipogenic lineage [15]. Currently, the term MSCs is used to describe a heterogeneous population of multipotential stem/progenitor cells commonly referred to as mesenchymal stem cells, multipotential stromal cells, mesenchymal stromal cells, and mesenchymal progenitor cells [16]. MSCs are in the spotlight as a potential cell-based therapy in orthopedics [17]. Although bone has the ability to self-regenerate, in some circumstances, depending on comorbidities, size of defects, and age, the ability can be reduced or even lost [12]. Moreover, changes in the mechanism of bone remodeling, maintained by a sensitive balance between bone resorption (osteoclasts activity) and new bone formation (osteoblasts activity), may cause certain diseases, such as osteopetrosis due to excessive bone formation or osteoporosis due to excessive bone resorption [10].

Currently, tissue engineering seems to be a promising subject of research related to cases of bone diseases in which the standard surgical procedures and pharmacological treatment have failed [18]. Nevertheless, the development of these bioengineering methods requires a complex, step-by-step approach, in which numerous variables have to be optimized. In particular, the main three components of bone engineering are (1) cells, (2) osteogenic factors, and (3) a scaffold. In the first step, it is necessary to conduct a preliminary but essential, in vitro optimization study in order to assess the source of the stem cells, choice of bioactive factors, and scaffold design. Next, before a clinical application in humans is viable, the in vitro optimized tissue engineering approach should be tested in an in vivo environment. Therefore, another issue to be considered is the most appropriate experimental animal model [19].

### 3.1. Sources and Biological Properties of MSCs for Bone Regeneration

A promising skeletal repair strategy in pathological bone disorders is to increase the number of osteogenic progenitor cells [20]. As described above, MSCs fulfil a key function in the processes of bone modeling, remodeling, and repair. They differentiate into the cartilage growth plate forming chondrocytes, which then gradually transdifferentiate into new bone-forming osteogenic cells during endochondral ossification. Alternatively, in the case of intramembranous osteogenesis, MSCs can directly differentiate into osteoblasts [6].

Cells with MSC characteristics can be isolated from adult and perinatal tissue sources [21,22,23]. Adherent cells, isolated from different tissue sources, should meet the minimal criteria of the MSC phenotype, such as the expression of the non-hematopoietic common markers CD73, CD90, and CD105 in over 95% of the cells, and the lack of expression of the hematopoietic and endothelial surface markers CD34, CD45, CD14 or CD11b, CD19 or CD79a, HLA-DR, and CD31, as defined by the International Society for Cellular Therapy (ISCT) [24]. These markers represent the accepted standards of MSC characteristics. However, controversies still exist regarding the ideal marker or set of markers, depending on the tissue sources of MSCs, culture conditions, and number of passages [22]. To date, bone marrow-derived MSCs (BM-MSCs) have been the most extensively characterized in terms of their phenotype and biological properties. Many studies on the biological characteristics of MSCs derived from other tissues are based on a comparative analysis with BM-MSCs [22,23,25]. In general, BM-MSCs meet the phenotypic criteria defined by the ISCT in terms of the classical positive (CD90, CD105, and CD73) and negative (CD34, CD45, CD14, CD79a, HLA-DR, and CD31) markers. In addition, BM-MSCs express other markers, including CD13, CD29, CD44, CD49, CD54, CD140b, CD146, CD276, and stage-specific embryonic antigen-1 (SSEA-1), but the expression of these markers is uncommon for MSCs derived from other tissue sources [21,26]. Studies showed that the presence of CD146 on BM-MSCs affects osteogenesis and angiogenesis [27]. Moreover, BM-MSCs are the most stable for CD146 expression during the subsequent passages compared to other sources of MSCs, including adipose tissue-derived MSCs (AT-MSCs) [22]. Although AT-MSCs meet the common criteria of the MSCs phenotype, they differ in CD34 expression during the early period of culture when the CD34 antigen displays a different level of expression [23,28]. Moreover, to distinguish AT-MSCs from BM-MSCs, two other markers, such as CD36 (GPIIIb) and CD106 (VCAM-1), are employed, because it was reported that AT-MSCs, in contrast to BM-MSCs, do not express CD106, but are positive for CD36 [28]. Comprehensive comparative studies performed on BM-MSCs and the cord blood-derived MSC (CB-MSC) phenotype revealed that among the 246 analyzed surface markers, both types of MSCs showed a high expression of 18 markers, including the classical ones (CD90, CD105, and CD73), as well as the alpha-smooth muscle antigen (SMA), CD13, CD140b, CD276, CD29, CD44, CD59, CD81, CD98, HLA-ABC, and vimentin. The presence of CD143 exclusively on BM-MSCs is suggested as a discriminating marker between adult and perinatal MSCs [23]. In addition to cord blood, MSCs can be obtained from other perinatal tissue sources, including the umbilical cord (UC-MSCs), placenta (PL-MSCs), amniotic fluid (AF-MSCs), and amniotic membrane (AM-MSCs). Although MSCs isolated from perinatal tissues have the characteristics of MSCs, they differ in the osteogenic potential [29,30]. Specifically, AM-MSCs and UC-MSC demonstrated a greater osteogenic differentiation capacity compared to MSCs isolated from other regions of perinatal tissues [30].

Furthermore, in addition to giving rise to the target tissue, MSCs also modulate the bone microenvironment, providing cytokines that support the vascularization of the new bone and facilitate the bone repair process [20]. MSCs exert a paracrine effect on the microenvironment by secreting various bioactive factors with an anti-inflammatory, immunomodulatory, trophic, proangiogenic, and pro-regenerative potential. Immunoregulation constitutes a crucial paracrine activity of MSCs, making them very special cells that affect not only immune cells, but also the microenvironment during the regeneration process. In response to inflammatory cytokines, such as interleukin-1 (IL-1), IL-2, IL-12, tumor necrosis factor-α (TNF-α), and interferon-γ (IFN-γ), MSCs secrete anti-inflammatory factors, including prostaglandin-2 (PGE-2), transforming growth factor-β1 (TGF-β1), IL-4, IL6, IL-10, and IL-1Ra, that stimulate tissue repair and modulate inflammation and immune response. The secretion of anti-inflammatory cytokines results in the downregulation of the function of the different immune cells related to innate and adaptive immunity (macrophages, natural killer cells, dendritic cells, T-lymphocytes, and B-lymphocytes), leading to a decrease in the inflammatory response. The increasing level of IL-4 and IL-10 promotes a shift in T-lymphocytes from the T-helper type 1 (Th1) to the Th2 phenotype and a shift in macrophage balance from the M1 (proinflammatory) to the M2 (anti-inflammatory) phenotype, thus inducing the anti-inflammatory milieu [16,31]. The trophic properties of MSCs are associated with the secretion of bioactive factors involved in cell proliferation and angiogenesis. MSCs produce TGF-α, TGF-β, hepatocyte growth factor (HGF), epithelial growth factor (EGF), insulin-like growth factor 1 (IGF-1), FGF-2, VEGF, angiopoietin-1 (Ang-1), and other growth factors and molecules that regulate cell proliferation and angiogenesis [31,32,33]. However, a screening of the MSC secretome revealed that BM-MSCs had the highest ability to secrete proangiogenic factors, such as IL-8 or VEGF, compared to AT-MSCs and skin-derived MSCs [22]. An increased production of IL-8 and VEGF by AT-MSCs was also reported in studies on the angiogenic activity of MSCs in a microgravity microenvironment [34]. Thus, the secretion of proangiogenic factors is a very desirable feature of MSCs, especially in the context of creating a living tissue using bone tissue engineering.

Interestingly, the differentiation potential and proliferation rate of MSCs may vary, depending on the tissue source [22,35]. In all likelihood, the specific niche in which they reside modulates the microenvironment and influences MSC properties [36]. Nevertheless, from numerous sources of MSCs, the most popular are bone marrow and adipose tissue, as the best known and well characterized [37]. Adipose tissue-derived MSCs can be isolated at relatively high density, in contrast to BM-MSCs. However, BM-MSCs and MSCs isolated from the human amnion show stronger osteogenic potential compared to AT-MSCs [29,38]. The procedures for obtaining MSCs from both bone marrow and adipose tissue are invasive, whereas MSCs originating from perinatal tissues, including the umbilical cord, placenta, amniotic fluid, and amniotic membrane, are available as medical waste. Among these, Wharton’s jelly seems to be a very good source of MSCs because of its ease of isolation and having no ethical concerns. It is worth mentioning that new sources of MSCs are currently considered for tissue regeneration, including muscles, skin, dental pulp, tendons, and the periodontal ligament [21,39]. Interestingly, in addition to the type of MSC source, the patient’s age and health are other important factors affecting the properties of the isolated MSCs [40].

Collectively, different sources of MSCs affect their biological properties and regenerative potential. Furthermore, in vitro cell culture can also change their ability to proliferate and differentiate. However, manipulating the culture conditions, such as introducing specific media supplements or hypoxia, may result in a more efficient MSC expansion and osteogenic differentiation [41,42].

#### Safety and Limitation of MSC Therapy

With over 25 years of history, MSC-based therapy has shown a very good safety profile. However, it is still employed as an experimental clinical procedure [16]. The reason lies in the biological diversity of MSCs, depending on their original tissue location, age of the donor, isolation method and expansion, and culture environment. All these factors affect the biological behavior of MSCs, making their in vivo activity difficult to predict.

The efficacy of MSCs-based therapy also depends on the delivery route. Intravenous infusion is the most common method of MSC administration. However, the limitation of this method is that a proportion of the transplanted cells are trapped within organs with a large capillary bed, especially in the lungs and liver, thus impairing the homing of infused MSCs to the target tissue; nevertheless, they are able to home to the injury site [39]. Moreover, the accumulation of MSCs in small capillaries carriers a risk of thromboembolic complications [43]. Experimental studies on the pig model have shown that intraarterial BM-MSC infusion is more effective in avoiding pulmonary BM-MSCs entrapment compared to intravenous infusion [44]. Delivery of MSCs to the site of tissue injury has a beneficial effect on the local anti-inflammatory response and directly affect damaged tissue repair. On the other hand, MSCs delivered locally have a restricted migratory potential, and their pro-regenerative activity may be limited to a small area of the damaged tissue.

It is also difficult to obtain sufficient numbers of MSCs for clinical application during in vitro manufacturing, especially in the early passages (up to the fifth passage). Long-term culture affects the biological potential of MSCs [22] and results in a decrease of proliferation and differentiation activity. Moreover, long-term culture of MSCs can increase the potential genetic instability of the cells and lead to a malignant transformation [39]. Therapeutic effectiveness is also related to the number of doses of MSCs when transplanted in allogeneic conditions. The administration of single dose of MSCs is safe and does not trigger the immune response; however, repeated doses of MSCs may induce the alloantibody production [45].

Standardization of the isolation methods and culture conditions and understanding the factors that underlie MSC biology should constitute important points for consideration before the use of MSCs in clinical practice. To date, MSC therapy has undoubtedly shown a favorable safety profile. On the other hand, long-term observations are necessary to assess the therapeutic effects of applied MSCs, including the adverse effects, in terms of cell sources, doses, and route of delivery.

### 3.2. Cytokines, Growth Factors and Signaling Pathways Enhancing Osteogenesis

**Figure 1:**
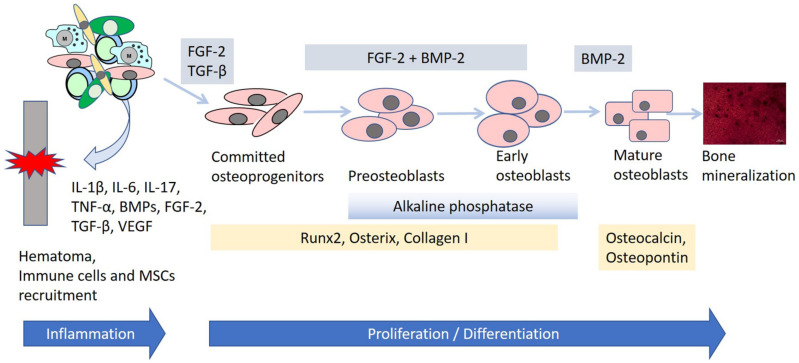
Schematic representation of the osteogenic differentiation of mesenchymal stem cells (MSCs) during bone regeneration. The first step of bone healing is the formation of fracture hematoma. The local hematoma attracts immune cells, creating an inflammatory microenvironment (IL-1β, IL-6, IL-17, TNF-α) and MSCs with an osteogenic and proangiogenic potential (TGF-β, BMPs, VEGF). Proliferation and osteogenic differentiation of MSCs is warranted by the simultaneous activity of FGF-2, TGF-β, and BMPs. BMPs increase the expression of osteogenic markers in MSCs, including the early osteogenic markers alkaline phosphatase, Runt-related transcription factor 2 (Runx2), osterix, and type I collagen, and the late markers osteopontin and osteocalcin. The bone mineralization image is taken from the authors’ own collection of the osteogenic differentiation of MSCs (Alizarin Red staining).

It is well known that bone fracture healing involves overlapping processes, i.e., inflammation, angiogenesis, and osteogenesis. Inflammation causes the secretion of various growth factors and cytokines, which in turn affect MSC recruitment and differentiation (Figure 1). Furthermore, these cytokines regulate the formation of vasculature, enabling bone remodeling [46]. In other words, osteogenesis requires not only cellular differentiation and tissue remodeling, but also appropriate molecular signaling. Therefore, it is crucial to understand how cytokines and growth factors enhance the effectiveness of bone repair and thus can promote particular approaches in bone tissue engineering [47]. The main molecular regulators of the bone healing cascade are bone morphogenetic proteins (BMPs), the fibroblast growth factor (FGF), transforming growth factor-β1, and vascular endothelial growth factor [3]. In addition, multiple signaling pathways regulate osteogenesis, including Wnt, Notch, parathyroid hormone (PTH), and hedgehog (Hh) [48,49].

BMPs, which were originally found in the extracts of demineralized bone, are a group of proteins belonging to the TGF-β superfamily [50]. Currently, these proteins are considered to be the most beneficial in the healing of large bone defects [3]. They are involved in embryogenesis, organogenesis, and cell proliferation and differentiation [51]. Their osteoinductive functions were discovered when they were found to induce de novo bone formation in demineralized bone [52]. They are responsible for MSC osteogenic differentiation, bone formation, and skeletal development. In particular, the BMP signaling pathway plays a crucial role in the differentiation of MSCs into the osteochondroprogenitor cells [53], after which they allow the differentiated osteoblasts to secrete the bone formatting matrix [54]. Moreover, BMPs have been shown to increase the expression of osteogenic markers in MSCs, including the early osteogenic markers alkaline phosphatase (Alp), Runt-related transcription factor 2 (Runx2), osterix (Osx), and type I collagen (ColI) and the late markers osteopontin (Opn) and osteocalcin (Ocl) [53,55,56,57]. Currently, BMP-2 (Medtronic) and BMP-7 (Stryker Biotech) are approved by the Food and Drug Administration (FDA) and available for clinical use in a recombinant form for bone fracture treatment and intervertebral disk regeneration enhancement [57].

FGFs regulate multiple processes of homeostasis and tissue development, including skeletal formation [58]. However, they do not directly influence osteogenic differentiation; rather, they modulate it, playing the role of an osteogenesis accelerator. They can stimulate osteoblast proliferation, promote differentiation into the osteogenic cell lineage, as well as induce angiogenesis [59]. Studies showed that FGF-2 and BMP-2 could act synergistically in bone regeneration, enhancing the effectiveness of bone formation [56,60,61].

TGF-βs are found in large amounts in the bone and cartilage. TGF-β1 stimulates bone growth and mineralization through the maintenance and expansion of MSCs, which then give rise to the osteoblastic lineage [62]. TGF-β signaling also enhances the proliferation of osteoprogenitor cells and their early osteogenic differentiation stages. Interestingly, interplay was found between the signaling of TGF-β and FGFs or BMPs in the bone [57]. For instance, TGF-β and FGF-2 stimulate osteoblasts proliferation, but on the other hand, inhibit alkaline phosphatase activity and mineralization. Consequently, it has been suggested that both of these cytokines can be potentially applied in tissue engineering for the induction of bone growth in vitro [63]. Furthermore, TGF-β1 strongly promotes BMP-2-induced osteogenic functions in bone formation in vitro [57].

VEGF is the most extensively explored angioinductive factor [46]. As bone is a strongly vascularized tissue, it requires re-vascularization after the fracture has occurred. Among others, osteoprogenitor cells, minerals, and signaling factors are thereby brought to the damaged area to promote the formation of new bone [3]. Some authors suggest that VEGF stimulates bone formation not only indirectly by promoting vascularization, but also directly by affecting osteogenesis through osteoblast and osteoclast attraction [47]. Studies have shown that a co-delivery of VEGF and BMPs may increase the efficiency of bone formation [64,65].

The Wnt signaling pathway is known to play a pivotal role in skeletal development and homeostasis, among others, through the promotion of osteoblast proliferation, differentiation, and maturation [66,67]. Wnt proteins bind to their receptors, the Frizzled and low-density lipoprotein receptor-related proteins (Lrp), which activates the main player of the pathway [68,69]. There are two categories of Wnt signaling pathways: canonical and non-canonical. In the canonical Wnt signaling pathway, the central activated protein is β-catenin. In the absence of Wnt ligand binding, β-catenin is destroyed by the protein complex through phosphorylation, ubiquitination, and degradation by the ubiquitin-dependent proteasomal system [70]. The canonical way inhibits the destructive proteasome complex, resulting in the translocation of β-catenin into the nucleus and the regulation of the Runx2 and Sp7/Osterix gene expression, which are involved in bone formation and differentiation. In turn, Runx2 and Sp7/Osterix positively regulate the gene expression of other osteogenic transcription factors, such as Alp, Opn, and Ocl [68]. It was reported that mice with an activated form of β-catenin in the osteoblasts and knockout of Axin2, one of the β-catenin destruction complex proteins, showed significantly increased bone healing and high bone mass [71]. The non-canonical Wnt signaling pathway, also called β-catenin-independent, uses Wnt5a or Wnt11 binding to a receptor complex and calcium signaling as the central mediator [72]. Studies also showed that Wnt5a-deficient mice had a reduced number of osteoblasts and low bone mass, suggesting that this non-canonical Wnt signaling pathway plays an important role in MSC differentiation into osteoblasts [73].

Another signaling pathway that directly affects osteoblasts and thus plays a significant role in bone tissue development is the Notch signaling pathway [74]. It is activated through the interaction between the Notch receptors and its ligands, resulting in the release of the Notch Intracellular Domain (NICD) and its translocation into the nucleus to activate the target genes [75]. It was demonstrated that the inhibition of the Notch signaling pathway in progenitor bone cells led to the reduction of bone marrow-derived MSCs and bone loss [76]. Moreover, the treatment of MSCs with Jag-1, one of the Notch signaling ligands, increased the expression of osteoblast-related genes: Alp and Bone Sialoprotein [77]. Another study showed that the Notch signaling pathway enhanced the osteogenic differentiation of MSCs in vitro and in vivo through the induction of BMP-9 signaling [78]. Interestingly, Lee et al. suggest that there is a crosstalk between the Notch and Wnt signaling pathways. Their study demonstrated that the regulation of osteoprogenitor cell proliferation during the formation of intramembranous bone was controlled by the Notch pathway, whereas the canonical Wnt pathway initiated the differentiation of osteoprogenitor cells [49].

The signaling pathway of the parathyroid hormone (PTH) is another example of a positive osteoblastogenesis regulator. The secretion of PTH occurs when there is a low level of calcium or calcitriol in the serum [79]. The PTH signaling pathway is activated through the binding between PTH and its receptor, which leads to the downstream signaling induction and activation of the cAMP-responsive element binding (CREB) [48]. In turn, the activated CREB positively affects the expression of osteogenic markers, such as Bmp-2, Ocn, and Bone Sialoprotein (Bsp), enhancing bone formation [80]. Xiao et al. presented results indicating that the crosstalk between FGF2 and Wnt signaling was required to mediate the maximal bone anabolic effects of PTH [81].

The last example of the signaling pathway enhancing osteogenesis concerns the Hedgehog (Hh). It is involved in BM-MSC differentiation into osteoblasts by affecting the expression of Runx2 and Osx [82]. Hh signaling also promotes the receptor activator of the NFkB ligand (Rankl) expression in osteoblasts by upregulating the expression of the parathyroid hormone related protein (PTHrP). As a result, RANKL regulates osteoclast differentiation and thus maintains homeostasis between bone formation and bone resorption [83]. Osteoblastogenesis in the endochondral skeleton is induced by the synergistic interactions between Hh and BMP [84]. Furthermore, studies found that crosstalk between the Hh and Wnt pathways regulated endochondral bone formation, cartilage development, and synovial joint formation [85].

### 3.3. Bone Scaffolds in Tissue Engineering

As previously mentioned, tissue engineering is a multidisciplinary field based on cell biology, molecular science, and biomaterial engineering. The third basic element in bone tissue engineering is the scaffold. Cells in the body grow in a three-dimensional environment, which enables them to interact with the extracellular matrix and other cells. Scaffolds in tissue engineering act as the extracellular matrix, supporting cell proliferation, adherence, differentiation, spreading, and communication [86].

#### 3.3.1. Scaffold Properties

For a safe and successful use in clinical settings, biomaterials for bone tissue engineering should exhibit several properties, such as biocompatibility, biodegradability, osteoinduction and osteoconduction, scaffold pore structure and grain size, and surface topography [1,87]. The scaffold should not stimulate the immunological response while being incorporated into the host tissue and should degrade into simpler substances that can be used by the body. Importantly, the level of degradation must be monitored and precisely matched to the level of bone regeneration [46]. The scaffold should be able to recruit osteoprogenitor cells to the fracture site, as well as induce the osteogenic differentiation of cells [87]. Another significant aspect are the well-defined structural properties of the scaffold, because they directly affect the cellular response. Scaffold porosity enables cell settlement and migration and the transport of nutrients and metabolites. Furthermore, it supports vascularization and production of the extracellular matrix [18]. Highly porous scaffolds are often used in bone regeneration to mimic the porosity of the trabecular bone [46]. Proper grain size provides adsorption sites for proteins and improves cell adhesion, proliferation, and differentiation [88]. In turn, surface topography ensures interactions between biomaterial and tissue [1]. Furthermore, a rough surface stimulates osteoblast-like cell spreading and proliferation [89]. Thus, the properties of the scaffold play a crucial role in regulation of biological responses [90].

#### 3.3.2. Scaffold Types

There are three main types of biomaterials: (1) bioceramics, (2) biodegradable polymers, and (3) composite biomaterials.

1. Bioceramics

Ceramic biomaterials are known for their high biocompatibility. The most commonly used bioceramic material is calcium phosphate (Ca-P) [3]. Among Ca-P ceramics, hydroxyapatite (HA) and tricalcium phosphate (TCP) are particularly interesting due to their similar compositions as natural bone [91]. Apart from effective biocompatibility, they also show high osteoconductivity and an ability to osseointegrate within the fracture site [92]. Furthermore, their biodegradation products are used in human metabolic pathways, enhancing cell activity and bone repair through the creation of an alkaline environment [93]. While HA is very advantageous in bone engineering because of its origin—it is the main mineral in natural bone and is thus highly osteoinductive—it is also very stable and hard to degrade in vivo. On the other hand, TCP is more degradable, and at the same time, it is also an effective bone biomaterial [94].

Currently, ceramics based on Ca-Si and bioactive glasses are studied extensively. This is due to the fact that Ca-Si ceramics have higher mechanical stability than Ca-P ceramics [91]. Ca participates in bone and blood vessel growth [95], whereas Si enhances bone calcification and density and prevents osteoporosis [96]. Bioactive glasses also have a great potential for bone repair through a rapid strong chemical binding with the bone tissue [97]. Furthermore, they are the only bioceramic material to date able to bond with both soft and hard tissues [91].

In general, ceramics are a good candidate for scaffolds used in bone tissue engineering because of their high bioactivity and biocompatibility. However, there are some limitations related to their low toughness and insufficient strength. For this reason, they are limited to load-free or low-load applications. To enhance the mechanical properties of bioceramics, studies are conducted on surface coatings, nanoscale second phase, and self-toughening methods [3,91].

2. Biodegradable polymers

Polymeric biomaterials can be categorized into natural and synthetic [98]. Biopolymers are known to support tissue growth and remodeling prior to their biodegradation [3]. Biodegradable polymers derived from plant and animal tissue, such as cellulose, collagen, and chitosan, are natural and characterized by good bioactivity, availability, cell affinity, non-toxicity, and a low risk of inducing an immune response [99].

An advantage of synthetic polymers is the possibility to modulate their surface properties and degradation degree through molecular design and synthesis [100]. Consequently, their mechanical properties and plasticity can be improved, in contrast to natural polymers [101]. The best known synthetic polymer is the poly(lactic acid) (PLA). It has been shown that PLA can be used as an effective biomaterial in bone repair due to its good biocompatibility, plasticity, and biodegradability and an ability to support osteoprogenitor cell adhesion and growth [102]. The poly(glycolic acid) (PGA) and poly(lactic-co-glycolic acid) (PLGA) are other synthetic biomaterials with good biocompatibility and biodegradability. The development of synthetic biomaterials combining good biological and mechanical properties allowed for PLA, PGA, and PLGA to be approved by FDA for clinical applications, such as bone scaffolds, surgical sutures, and injection capsules [91]. However, the most favorable biomaterial for bone tissue engineering is polycaprolactone (PCL), which is also FDA-approved. Compared with other synthetic biodegradable polymers, PCL shows better mechanical properties [3].

Although polymer scaffolds are widely used in bone tissue engineering, there are some disadvantages, such as weak mechanical properties, risk of deformation, and problems with a strong integration with the bone. In general, synthetic polymers have poor bioactivity and thus poor cell adhesion, whereas natural polymers are difficult to process and control in terms of their degradation and show poor thermal stability [103]. However, these limitations can be addressed through the use of the nanoscale second phase method, as previously mentioned for ceramic biomaterials [104].

3. Composite biomaterials

The mechanical properties of biodegradable scaffolds can be improved through the use of composites, consisting of ceramics combined with polymers. Consequently, biodegradable scaffolds possess the advantages of both types at once, such as improved biocompatibility and bioactivity, mechanical toughness, host–implant interactions, and load-bearing capabilities [105]. Examples of effective composite biomaterials include PLGA and PCL combined with TCP or HA. They have the high bioactive potential of ceramics, promoting the formation of mineralization sites while allowing for the controlled degradation kinetics of the polymers. Moreover, they maintain an appropriate balance between strength and toughness [106].

#### 3.3.3. 3D Printing of Bone Scaffolds

The success of bone tissue engineering-based treatment depends, to a large extent, on scaffolds with appropriate features, such as structure, shape, and chemical, physical, and biological properties [107]. Manufacture of traditional scaffolds relies on the following techniques: lyophilization, solvent casting, electro- and wet spinning, porogen leaching, and gas foaming. Although these methods are widely used in tissue engineering, they have certain limitations, such as a long manufacturing time, use of toxic organic solvents, and low reproducibility [108]. Many studies have shown that one of the most important aspects of tissue engineering are the interactions between the cells and the material. For instance, the size of pores should be optimized to help the cells migrate, proliferate, and differentiate [109,110,111]. A customized scaffold with the anatomical shape of the bone defect and highly desirable characteristics for bone regeneration can be produced via three-dimensional (3D) printing. The method can create novel scaffolds with favorable architecture, mechanical strength, wettability, and cellular response [107]. Moreover, 3D modelling and printing are very simple, fast, and accurate, allowing for many tests to be conducted on biomaterials [108].

Three-dimensional printing, also called the additive manufacturing method, is based on piece-by-piece building. The designed object can be manufactured without any loss of material [112], which is the opposite of subtractive methods, in which superfluous material is removed until the appropriate shape is achieved [108]. Before a 3D printer can start creating a solid, complex object layer-by-layer, a computer-aided design (CAD) file must be prepared first [113]. Computer modeling of a patient-specific bone scaffold is divided into two steps: data acquisition and model generation. It is important that these steps be completed with appropriate care due to variations in bone anatomy between the patients. These steps directly affect the quality of the final medical part to be replaced [114]. The anatomical data about the shape and size of a bone defect can be acquired via computed tomography (CT) or magnetic resonance imaging (MRI) [115]. Next, a customized scaffold geometry is obtained using CAD software [114].

The most commonly used type of material in 3D printing are polymers. This is due to their mechanical properties, surface chemistry, and topography. However, other materials are also widely used, such as ceramics and metals [107,116]. In recent years, there has been a rapid rise in the popularity of biocompatible materials, as well as complex 3D products with living cells. These are created with bioprinting and are able to mimic the biological functions of their native tissue analogues [117]. Over the last two years (2019–2020), the number of publications on 3D printing in bone regeneration has skyrocketed, with about 1400 published papers and the number continuing to grow (www.pubmed.ncbi.nlm.nih.gov accessed on 24 July 2021).

Although 3D printing has gained great attention and is widely used in bone tissue engineering, it has some limitations. For example, it is crucial to provide the scaffold with vascularization-enabling oxygen and enough nutrient transportation to support bone regeneration. However, very few scaffolds are designed to regenerate the defected bone with the required vascularization [107]. Three-dimensional printing only considers the initial structure of the designed object and assumes it to be static and inanimate. Interestingly, a new method has been developed to overcome this problem, namely, 4D printing. It is a relatively new technique based on smart biomaterial integration, allowing the shape and functionality transformation of the scaffold to change when exposed to external stimuli [118].

### 3.4. Animal Models for In Vivo Studies

To predict the clinical efficacy of novel bone treatment techniques based on tissue engineering, it is necessary to provide proof of concept in an animal model. Although in vitro experiments bring an extensive insight into cellular processes and molecular mechanisms, in vivo studies can mimic the many integrated pathways in certain pathological conditions at the level of the entire body [119]. The most commonly used animal models are rodents, for instance mice or rats. This is due to low costs and ease of handling and breeding to assess the reproducibility of an experiment. Furthermore, the mouse is a well-characterized animal, and there are many molecular tools, antibodies, and other materials available for the evaluation of research performed on this animal model. With experimental manipulation, they are also easily adaptable to pathological conditions [120,121]. However, there are many restrictions associated with the use of rodent models, such as a lack of cortical remodeling and limited trabecular bone content [122]. Naturally, the size of the bone defect also plays an important role, as it influences the mechanism of bone regeneration. The biomechanical conditions of human large defects cannot be adequately simulated in small animal models [123]. Therefore, use of large animal models, such as sheep or dog, provides a more advantageous ratio between their body weight and bone size and structure and the same parameters in humans [121]. Moreover, the immune system of large animals is more similar to the human one than that of small animals. This is especially significant in research on the impact of immunogenic factors and cells on bone healing [124]. The animal’s size and anatomy should be as similar to human as possible for the purposes of investigating bone healing processes [125]. Translational proof of concept needs to be provided in a large animal model; however, initial tests on a small animal model are acceptable [119]. Nevertheless, the FDA often finds it necessary to test a new bone therapy in both a small and large animal model before approving it for clinical trials. For instance, this applies to research on osteoporosis [126]. In general, when selecting an animal model for research on bone tissue engineering, first and foremost, it is important to clearly identify the issue to be resolved. Some of the criteria that should be considered are functionality, mechanical testing, histology, and biochemical and molecular assays. They will affect the complexity of the experiment design. As there are many problems to be solved in the bone engineering field, the solutions can be achieved using different animal models [127].

### 3.5. Preclinical Studies

When developing MSC-based therapies, the extracellular environment has been indicated as the crucial factor that affects MSC survival, proliferation, biologically active growth factor and cytokine secretion, and differentiation [128]. Biomaterials can be used to culture conditions resembling the natural cell microenvironment in the native tissue. However, only in vivo studies using an appropriate animal model mimicking the clinical setting can help to assess the efficacy of the engineered tissue. The regeneration of a functional bone tissue requires an in vivo environment with a complex of biological and biomechanical properties. After this necessary in vivo phase, it may be possible to perform human clinical trials [19]. This section presents examples of successful in vivo studies on bone regeneration performed over the last decade (2010–2020) using MSCs and various types of scaffolds (Table 1).

In the in vivo studies on scaffolds and MSCs in bone regeneration presented above, the most commonly applied scaffolds were bioceramics (especially β-TCP) and biodegradable polymers. Masaoka et al. prepared composites of β-TCP and monkey bone marrow-derived MSCs for large bone defect treatment in a non-human primate model. They succeeded in the reconstruction of 5-cm-long bone defects using β-TCP scaffolds with or without BM-MSCs in a monkey model. However, only three of the nine cases treated with the scaffold alone exhibited bone regeneration. In contrast, scaffolds with cell treatment led to successful bone reconstruction in five out of the seven monkeys [136]. Another study based on β-TCP was conducted for calvarial bone defect repair in rats. Mesenchymal stem cells from rat bone marrow were differentiated into osteogenic cell sheets and induced endothelial-like cells. Then, a vascularized cell sheet was formed by means of induced endothelial-like cell cultivation on an undifferentiated MSC sheet. Together, the osteogenic cell sheet and the vascularized cell sheet formed a biomimetic periosteum (BP), which was then wrapped onto a β-TCP scaffold. As control groups, a β-TCP scaffold with autologous periostea and a β-TCP scaffold alone were used. The results showed promoted formation of blood vessels and new bone tissue formation in the BP/β-TCP scaffold treatment as well as the β-TCP scaffold with autologous periostea treatment [139]. Furthermore, Lin et al. conducted a study on the reconstruction of bone damage involving a loss of periosteum using a TCP scaffold with human MSCs in the pig. The results showed that MSCs and TCP synergistically enhanced the bone healing effect and increased lamination and vessels [141]. In turn, Chu et al. treated critical size bone defects in goat tibia of 30 mm with MSCs and a β-TCP scaffold. Six months after transplantation, the repair effect was significantly higher in the MSCs/ β-TCP group compared to the β-TCP-only group [140].

Another example of a bioceramic scaffold is hydroxyapatite. Yea et al. investigated a hydroxyapatite-gradient scaffold (HA-G) isolated from adipose tissue with MSCs derived from the umbilical cord (UC) on the gradient structure of the rotator cuff tendon-to-bone interface (TBI) regeneration in a rat model. The study demonstrated the formation of tendon, cartilage, and bone matrices in rats treated with UC-MSCs and an HA-G scaffold. Moreover, the regeneration of the rotator cuff TBI in the rat model was similar to the normal TBI when comparing histological and biomechanical properties [143].

The PLLA scaffold belongs to the second category of scaffolds—biodegradable polymers. It was successfully used to treat cranial bone defects in rats. When MSCs were pre-seeded onto a scaffold and cultured in an osteo-lineage induction medium prior to the transplantation, the highest osteogenic ability of the 3D construct was observed, compared to an injection of MSCs into a scaffold during surgery [129]. In another study, a PEG/PLA scaffold with MSCs was transplanted into the thigh muscle pouches of rats, and the physiological characteristics of the surrounding tissues were evaluated. The results showed a very good osteogenic potential of the MSCs-scaffold construct in vitro and good biocompatibility in vivo. This makes the construct a very promising tool for bone tissue engineering [130]. Another study compared the use of PGA alone and PGA with autologous BM-MSCs in the rabbit model of the infraspinatus tendons defect. Sixteen weeks following implantation, the tendon maturing score and a mechanical analysis results showed higher values in the PGA-MSC-treated group than in the PGA-only treated rabbits [131]. Liang et al. used a PLGA scaffold with osteogenic-induced AT-MSCs to treat a spine defect. Bone formation occurred between two and four weeks after the MSCs-scaffold construct implantation. However, a second bone formation occurred in the group treated with the osteogenic-induced AT-MSCs and the scaffold and not in the group without the osteogenic induction [132]. Zhang et al. efficiently treated arthritis rats with nanofiber PLGA and BM-MSCs. The results revealed that the MSCs-scaffold construct suppressed bone destruction and arthritis. Moreover, in vivo MSC tracing demonstrated that they remained within the scaffold and did not migrate to other organs [133]. A segmental tibial bone defect of 3.5 cm in sheep was treated with PLLA-PCL. Twelve weeks post implantation, the scaffold alone and scaffold combined with skeletal stem cells (SSCs) enhanced bone regeneration. However, significant enhancement was observed only for the scaffold-SSCs group. Therefore, cell therapy combined with a scaffold can promote bone regeneration in a critical-sized bone defect compared to a scaffold alone [138].

A group of scaffolds that is widely used in pre-clinical studies are composite biomaterials. A PCL-HA with CaP scaffold was used to treat critical-sized segmental tibial bone defects in sheep. Interestingly, cell therapy was not applied together with the scaffold immediately after the defect creation. Instead, allogenic bone marrow stem cells were injected four weeks after the scaffold implantation in a post-inflammatory stage. This delayed cell injection significantly improved bone regeneration compared to scaffold-preseeded cell construct and scaffold-only groups [134]. In another study, a PLA-HA scaffold loaded with bone marrow-derived MSCs and induced membrane (IM), which provided growth factors, was used to regenerate large radial defects in rabbits. The results showed the best bone repair and reconstruction effect in the group treated with PLA-HA combined with IM and MSCs compared to PLA-HA alone or PLA-HA with IM [142].

The last two examples are based on the use of biological composites with progenitor cells. Yoon et al. investigated the osteogenetic effect of albumin scaffold derived from canine serum (ASA) and MSCs isolated form canine adipose tissue in segmental bone defects. The animals were treated with the ASA scaffold alone or with MSCs and the ASA scaffold including β-TCP with or without MSCs. Sixteen weeks after transplantation, the ASA scaffold with MSCs accelerated new bone formation significantly higher than the other groups [135]. In another in vivo study, a coral scaffold with bone marrow-derived MSCs and a low dose of BMP-2 was injected into 25-mm-long metatarsal bone defects in the sheep model. The most successful results were observed for the group treated with the coral scaffold-MSCs and BMP-2 compared to scaffold-BMP-2 or scaffold-MSCs alone [137].

## 4. Clinical Trials

Preclinical animal studies have shown a beneficial effect of MSCs-scaffold treatment in orthopedic disorders [144]. However, when creating experimental preclinical models, researchers need to remember that various species differ biologically between one another, and the results of animal experiments often depend on the choice of the proper animal model [145]. Nevertheless, animal models are essential for the bench-to-bedside translation of new treatment methods. Preclinical data are reproducible and translatable into clinical use only if animal experiments are properly designed [146]. There are only few publications from the last ten years (from 2010 to 2020) reporting the results of clinical trials based on scaffolds and MSCs used to repair damaged bone tissue (data from www.clinicaltrial.gov and www.pubmed.ncbi.nlm.nih.gov). This is likely due to flawed preclinical research validation, which is crucial to bridge the translational gap to the clinic [146]. In this section, selected examples of human trials using scaffold/MSC constructs for bone regeneration are introduced (Table 2).

The first example of a clinical study was conducted by Yamasaki et al. They have examined the transplantation effectiveness of interconnected porous calcium hydroxyapatite (IP-CHA) and bone marrow-derived mononuclear cells (BMMNCs) on early bone repair in femoral head osteonecrosis. Twenty-two patients (30 hips) with a mean age of 41 years (18 to 64) were studied. The control group consisted of eight patients (nine hips), who received a cell-free IP-CHA implantation. After a mean follow-up of 29 months (19 to 48), the size of the osteonecrotic lesion decreased in the IP-CHA- and BMMNCs-treated group, whereas subtle bone hypertrophy and a severe collapse of the femoral head was observed in the control group [147].

Jäger et al. investigated the augment bone grafting potency of a commercially available collagen sponge (Col) (Orthoss^®^, Fa Geistlich, Wolhusen, Switzerland) or bovine hydroxyapatite (HA) (Gelaspon^®^, Fa. Chauvin Ankerpharm GmbH, Berlin, Germany) with 8 mL of bone marrow aspiration concentrate (BMAC). All 39 patients between the ages of 4 and 87 years with local bone defects larger than 1 cm × 1 cm (length × width) showed new bone formation during follow-up. However, bone formation appeared earlier in the HA group (6.8 weeks), and complete bone healing was achieved after 17.3 weeks, in contrast to the Col group (13.6 weeks) with bone healing completed after 22.4 weeks [148].

Cuthbert et al. used induced membrane, which was a rich source of MSCs, to treat eight patients with critical-sized bone defects (mean size 36.25 mm) and a mean age of 60 years (between 18 and 80). They compared 1-cm^2^ biopsy samples after membrane formation and healthy diaphyseal periosteum. The IM had a cellular composition and molecular profile resembling periosteum, which facilitated the repair of the large bone defects [149].

Another study investigated the long-term efficacy and safety of BM-MSCs expanded ex vivo with autologous fibrin clots for the treatment of upper limb atrophic pseudarthrosis. Eight patients with a mean age of 44 years (between 18 and 73), who had undergone at least one unsatisfactory surgical intervention, were selected for the implantation of an autologous MSC/fibrin scaffold construct. The study relied on: use of cells, serum for ex vivo cell culture and scaffold components (an entirely autologous context); reduced cell expansion ex vivo; and short-term osteoinduction of MSCs before implantation. On the day of the surgery, 0.5 × 10^6^–2.0 × 10^6^ MSCs were resuspended in 2 mL of autologous plasma and implanted with a fibrin clot at the site of the lesion. After short- and long-term follow-ups (mean: 6.7 and 76.0 months, respectively), healing was evaluated radiographically. In all cases, positive clinical outcomes were shown with recovered limb function. The study demonstrated that the minimal MSC expansion ex vivo and short-term osteoinduction reduced the risk of an uncontrolled proliferation of the transplanted cells and, consequently, the implant overgrowth [144].

Šponer et al. compared the healing efficacy of femoral defects following the implantation of ultraporous β-tricalcium phosphate alone (nine patients, control group) or β-tricalcium phosphate with expanded 15 ± 4.5 × 10^6^ autologous bone marrow-derived MSCs (9 patients, trial group). Radiography and DEXA (bone density) scanning were performed 6 weeks and 3, 6, and 12 months post operation. In all nine patients of the trial group, trabecular remodeling was found, whereas in the control group, only in one patient [150].

The another example of a clinical study is registered in the Chinese Clinical Trial Registry and was executed from June 2013 to October 2016. Forty-two patients requiring bone graft received SECCS-based treatment. SECCS is a stem cell screen–enrich–combine (-biomaterials) circulating system, designed by Zhuang and his colleagues. This innovative system can process patients’ bone marrow cells and beta-tricalcium phosphate (β-TCP) granules to produce MSC/β-TCP composites in 10–15 min. Patients with a bone defect aged between 15 and 65 years were treated surgically with MSC/β-TCP composites. The full bone healing, including bone union observed in lateral and anterior–posterior radiography within nine months, was successful in all patients. The mean healing time for nonunion, fresh fracture, and other patients was 6.29, 3.12, and 4.72 months, respectively. The results showed that SECCS, which peri-operatively produces a bioactive composite of MSCs and β-TCP without in vitro culture, may represent a low cost and safe method for bone repair [151].

The same team evaluated the clinical efficacy of MSC/β-TCP prepared with the SECCS in 39 patients suffering from depressed tibial plateaus fractures. Sixteen patients were treated with MSC/β-TCP composites, whereas another 23 patients only with β-TCP. Eighteen months post MSC/β-TCP transplantation, new bone formation rate was significantly higher than in the patients treated only with β-TCP. The average new bone ratio in the first group was 91.9 ± 4.8%, and in the β-TCP-treated group, it was 21.9 ± 12.2% (W = 231.0; *p* < 0.01). Two years post implantation, MRI analysis revealed that the grafted composite had been replaced by new well-integrated autologous bone. The Lysholm score assessed the functional recovery of the patients. After two years post implantation, 15 of 16 patients treated with MSC/β-TCP (93.8%) and 14 of 23 patients treated with β-TCP alone (60.9%, *p* = 0.028) achieved excellent or good recovery. The MSC/β-TCP composite produced using the SECCS method was effective in the treatment of depressed tibial plateau fractures, promoting osteogenesis and improving joint recovery [152].

Another clinical study, conducted in Australia, involved cranial reconstruction using allogeneic mesenchymal stromal cells. MSCs were seeded on a ceramic carrier and a polymer scaffold to design a tissue-engineered construct. Three patients with cranial defects less than 80 mm in diameter underwent a baseline fine-slice CT to virtually reconstruct the bone defect and create a virtual 3D skull model. Then, a 3D printing method was used to produce the reconstructed surfaces of the defect, consisting of two polymer meshes corresponding to the skull interna and externa. The MSCs were isolated from the bone marrow of healthy donors aged between 18 and 40 years. The operation procedure was as follows. Firstly, the inner polymer mesh was placed, then the MSC-loaded granules, and finally, the outer mesh. The patients were followed up on 3, 6, and 12 months after surgery for visual cosmesis inspection. All patients displayed excellent initial cosmesis without any complications. Post-operative CT scans were conducted on day 1 and 3 and after 12 months to assess bone formation. Analysis of CT data showed good cranial contour restoration, which was maintained between 3 and 6 months post transplantation. However, there was evidence of construct resorption in all patients between 6 and 12 months. The continuously pulsating environment likely caused the lack of construct rigidity and therefore prevented solid bone formation. Nevertheless, a customized allogenic MSC-bone engineering construct for cranial reconstruction can be produced using computer modeling and tissue engineering. It is crucial to investigate constructs with appropriate rigidity to reconstruct bone defects, which can be achieved using 3D printing [153].

There are also studies evaluating bone formation using MSC-scaffold composites in oral and maxillofacial bone defects. Gjerde et al. from the University of Bergen conducted a clinical trial on 11 patients aged between 52 and 79 years with severe mandibular ridge resorption. The patients were treated with bone marrow-derived MSCs loaded on biphasic calcium phosphate (BCP) granules, implanted in the area of the resorbed alveolar ridge. New bone formation was assessed 4–6 months after healing. X-ray analysis showed a significant total bone volume increase. During implant installation in the newly regenerated area, bone was biopsied for μ-CT and histology to evaluate the formation of mineralized tissues. Successful ridge augmentation and new bone formation adequate for the installation of a dental implant were observed in all patients. During the first 12 months after the dental implant installation, Osstell values (measuring implant stability) increased for all study participants. This clinical trial showed that MSC- and BPC-based treatment of the alveolar ridge is safe, feasible, predictable, and could be considered as a less invasive approach to the reconstruction of maxillofacial bone defects than the current gold standard, which is autologous bone grafting [154].

The last example presents an early efficacy of the long bone delayed and non-union treatment with autologous bone marrow-derived MSCs and scaffold composed of 80% β-TCP and 20% HA. Twenty-eight participants, mean age 39 ± 13 years, with tibial (13 patients), femoral (11), and humeral (4) non-unions that occurred at a mean 27.9 ± 31.2 months prior recruitment, underwent surgical implantation. Bone healing efficacy was reported with clinical and radiological consolidation 3, 6, and 12 months after surgery and CT sections imaging 3 and 6 months after surgery. Moreover, in two cases, bone biopsies were performed during screw removal. Clinical consolidation results using the visual analogue scale (VAS) were as follows: 3 months, 24/28 patients (85.7%); 6 months, 24/27 (88.9%); and 12 months, 25/25, after completed follow-up. Radiological healing rate was 25% (7/28 patients) after 3 months, 67.8% (19/28) after 6 months, and 92.8% (26/28) after 12 months. Bone formation surrounding the bioceramic scaffold was confirmed with bone biopsies after 8 months [155].

## 5. Conclusions

Bone tissue engineering constructs based on a biomaterial scaffold and MSCs are undoubtedly a promising alternative to standard bone graft. Although MSCs are known to play a crucial role in bone repair process, there are still some factors and pathways to be fully understand and optimized. Mesenchymal stem cells participate in bone regeneration not only through direct differentiation into osteogenic progenitors, but also through paracrine activity by secreting a variety of cytokines and growth factors. Bioactive factors secreted by MSCs exert an anti-inflammatory and immunomodulatory effect on effector immune cells and modulate the microenvironment of the injured tissue.

Clinical use of an MSC-scaffold construct requires standardized MSC sources, osteogenic signaling factors, as well as scaffold design. MSCs isolated from different tissues are not universal, and for the purposes of bone repair, should be selected based on their osteogenic potential (e.g., BM-MSCs or UC-MSCs). MSCs alone are unable to cover large bone defects. However, modern technologies, such as biomaterials and 3D printing scaffolds with a proper structure employed in bone substitute engineering can support the osteoinductive properties of the applied MSCs. Innovative biomaterials used in tissue engineering for bone regeneration should be biocompatible and biodegradable and should fulfil specific biological properties to allow MSC adhesion, proliferation, and osteogenic differentiation in the injured bone area. It is also necessary to develop an appropriate preclinical animal model to assess the best therapeutic approach. Small animal models are useful for investigating the bone-related mechanism of healing. However, only large animal models are absolutely essential to mimic human clinical settings. Successful preclinical results enable the final step of bone tissue engineering development and application in clinical trials.

## Figures and Tables

**Table 1:** In vivo studies using MSC-based therapies with scaffolds for bone regeneration.

Animal	Cells (Suspension)	Scaffold	Treated Side	Results	References
Rat	Human BM-MSCs (2 × 10^6^ cells/mL)	PLLA	Cranial bone defect	Pre-seeding an MSCs-scaffold construct leads to a higher osteogenic capacity than for MSCs injected into a scaffold during surgery.	[129]
Rat	BM-MSCs	PEG/PLA	Thigh muscle pouches	An MSCs-scaffold construct had an excellent osteogenic potential in vitro and a good biocompatibility in vivo.	[130]
Rabbit	BM-MSCs	PGA	Defect of infraspinatus tendons	16 weeks after implantation, mechanical analysis and the tendon maturing score showed higher values in the MSC-scaffold treated group than in the PGA-only treated rabbits.	[131]
Rat	AT-MSCs (10^4^ cells per scaffold)	PLGA	Vertebral body of the spine defect	Between 2 and 4 weeks after MSC-scaffold construct implantation, bone formation occurred. However, in the group treated with osteogenic-induced AT-MSCs and a scaffold, a second bone formation occurred, contrary to the non-induced group.	[132]
Rat	Human BM-MSCs (2 × 10^4^ cells/cm^2^ or 2 × 10^5^ cells/cm^2^ of scaffold)	nano-fiber PLGA	Collagen-induced arthritis	An MSCs-scaffold construct suppressed bone destruction and arthritis in rats.	[133]
Sheep	BM-MSCs (100 × 10^6^ cells)	PCL-HA + CaP	Segmental tibial bone defect	For a delayed injection of BM-MSCs into a scaffold, 4 weeks after biomaterial implantation biomechanical testing and micro-CT analysis showed improved bone regeneration compared to previously-seeded PCL-HA-cell construct or scaffold-only group.	[134]
Canine	AT-MSCs (1 × 10^6^ cells/50 µL PBS)	(1) autologous serum-derived albumin (ASA) scaffold, (2) ASA + β-TCP	Segmental ulna bone defect	16 weeks post-implantation, radiograph and histomorphometric analysis showed the most extensive new bone formation in ASA with AT-MSCs compared to untreated, ASA-only, and ASA+β-TCP with or without AT-MSCs.	[135]
Monkey	BM-MSCs (1.3–4.1 × 10^6^/mL)	β-TCP	Segmental femoral bone defect	12 weeks after transplantation, β-TCP + AT-MSCs treatment led to a higher success rate of bone regeneration compared to β-TCP treatment alone.	[136]
Sheep	BM-MSCs (10^7^ cells)	coral scaffold	Long metatarsal bone defect	4 months post implantation, micro-CT and histological analysis showed better bone formation in the group treated with the construct scaffold + BMP-2 + BM-MSCs compared to scaffold + BMP-2 or scaffold + BM-MSCs.	[137]
Sheep	BM-MSCs (10^7^ cells)	PLLA-PCL	Segmental tibial bone defect	12 weeks after implantation, significant bone regeneration was confirmed with micro-CT, mechanical testing and histological analysis in the group treated with PLLA-PCL + BM-MSCs compared to PLLA-PCL-only and untreated group.	[138]
Rat	BM-MSCs, osteogenic and endothelial differentiated BM-MSCs (5 × 10^4^ cells/cm^2^ BM-MSCs sheet)—biomimetic periosteum (BP)	β-TCP	Calvarial defect	8 weeks post-surgery, micro-CT and histological analysis showed better new bone formation in β-TCP + BP and β-TCP + autologous periosteum groups than in the control groups.	[139]
Goat	BM-MSCs	β-TCP	Critical size bone defects in tibia	6 months after operation X-ray, micro-CT and histological analysis showed that the defect treatment using β-TCP + BM-MSCs was significantly superior to that using β-TCP alone.	[140]
Pig	Human AT-MSCs	TCP	Segmental long bone defect	8 and 12 weeks after reconstruction, radiographic images and pathological sections analysis showed that TCP + human AT-MSCs promoted bone healing.	[141]
Rabbit	BM-MSCs	PLA-HA	Radius long bone defect	8, 12, and 16 weeks post transplantation, micro-CT, X-ray and histological analysis showed enhanced bone reconstruction in PLA-HA + BM-MSCs combined with induced membrane group compared to the other groups.	[142]
Rat	Human UC-MSCs (2 × 10^5^ cells)	HA-G	Tendon-to-bone interface	After 8 weeks, histological and biomechanical evaluation showed that the total regeneration score was significantly higher in the HA-G + UC MSC group compared to the other groups.	[143]

Abbreviations: ASA—autologous serum-derived albumin, AT-MSC—adipose tissue-derived mesenchymal stem cell, BM-MSC—bone marrow-derived mesenchymal stem cell, BP—biomimetic periosteum, CaP—calcium phosphate, HA—hydroxyapatite, HA-G—hydroxyapatite-gradient scaffold, PCL—polycaprolactone, PEG—poly(ethylene glycol), PGA—polyglycolic acid, PLA—polylactide, PLGA—poly(lactide-co-glycolide) acid, PLLA—poly (l-lactic acid), UC-MSC—umbilical cord-derived mesenchymal stem cell, β-TCP—β-tricalcium phosphate.

**Table 2:** MSC-based therapies with scaffolds for the repair of bone defects in clinical trials.

Study Number	Disease	Cells (Suspension)	Scaffold	Patients (Groups)	Results	References
Not reported	Osteonecrosis of the femoral head	BMMNCs (1 × 10^9^ cells in 40 mL)	IP-CHA	30 patients: 8 patients treated with cell-free IP-CHA (control group) and 22 patients with IP-CHA + BMMNCs	29 weeks after surgery in the IP-CHA- and BMMNC-treated group, the osteonecrotic lesion decreased in size. In the control group, a severe collapse of the femoral head occurred in 6 patients.	[147]
Study #3096 Ethics Committee of the Heinrich Heine University Duesseldorf	Local bone defects larger than 1 cm × 1 cm	BMAC (8 mL)	Col or HA	39 patients: 12 patients treated with Col + BMAC and 27 patients with HA + BMAC	New bone formation was observed in all treated patients; however, it appeared earlier in the HA group (6.8 weeks) compared to Col (13.6 weeks).	[148]
Not reported	Critical size bone defects	IM as a complex cellular scaffold (rich source of MSCs)		8 patients	Cellular composition and molecular profile of IM-promoted large defect repair.	[149]
3766/2012 Comitato Etico Sperimentazione Farmaco CESF, Azienda Ospedaliero-Universitaria Pisana, Pisa, Italy	Upper limb atrophic pseudarthrosis	BM-MSCs (0.5 × 10^6^ –2.0 × 10^6^ cells in 2 mL of autologous plasma)	Autologous fibrin clots	8 patients	In all patients, recovery of limb functions was observed.	[144]
EudraCT number 2012-005599-33 EU Clinical Trials Register	Femoral defects	BM-MSCs (15 ± 4.5 × 10^6^ cells in 1.5 mL)	β-TCP	18 patients: 9 patients treated with β-TCP alone (control group) and 9 patients with β-TCP + BM-MSCs	12 months after surgery, in all 9 patients treated with β-TCP and BM-MSCs, trabecular remodeling was detected, and in the control group, only in one patient.	[150]
ChiCTR-ONC-17011448 Chinese Clinical Trial Registry	Non-unions and others	BM-MSCs	β-TCP	42 patients	In all patients, radiography showed full bone healing after 9 months.	[151]
2017-385-T282 Shanghai Jiao Tong University Affiliated Ninth People’s Hospital Medical Ethics Committee	Depressed tibial plateaus fractures	BM-MSCs	β-TCP	39 patients: 23 patients treated only with β-TCP (control group) and 16 patients with β-TCP + BM-MSCs	Excellent or good recovery was observed 2 years post transplantation in 15 of 16 patients treated with MSCs/β-TCP and in 14 of 23 treated with β-TCP alone.	[152]
EC2012/047 Royal Perth Hospital Ethics Committee	Cranial defects	BM-MSCs (min. 0.5 × 10^6^ cells per ml of scaffold granules)	β-TCP	3 patients	Between 3 and 6 months post transplantation, good cranial contour restoration was maintained in all three patients. However, between 6 and 12 months, there was evidence of construct resorption.	[153]
EudraCT, 2012-003139-50 EU Clinical Trials Register	Severely atrophied mandibular bone	BM-MSCs (20 × 10^6^ cells/1 cm^3^ of scaffold)	BCP	11 patients	In all patients, successful ridge augmentation and new bone formation of a dental implant were observed.	[154]
EudraCT, 2011-005441-13 EU Clinical Trials Register	Long bone delayed and non-unions	BM-MSCs	BCP	28 patients	3 months after surgery, radiological consolidation amounted to 25.0% (7/28 cases), after 6 months, 67.8% (19/28 cases), and after 12 months, 92.8% (26/28 cases).	[155]

Abbreviations: BCP—biphasic calcium phosphate, BMAC—bone marrow aspiration concentrate, BMMNCs—bone-marrow-derived mononuclear cells, BM-MSCs—bone marrow derived mesenchymal stem cells, Col—collagen sponge, HA—hydroxyapatite, IM—induced membrane, IP-CHA—interconnected porous calcium hydroxyapatite, β-TCP—β-tricalcium phosphate.

## Data Availability

Not applicable.
